# Food insecurity in Norway: A cross-sectional study among patients visiting their general practitioner

**DOI:** 10.1177/14034948241278781

**Published:** 2024-09-26

**Authors:** Noemi Cioffi, Esperanza Diaz, Elisabeth M. Strømme, Bjørn Bjorvatn, Thomas Mildestvedt, Lars T. Fadnes

**Affiliations:** 1 Department of Global Public Health and Primary Care, Faculty of Medicine, University of Bergen, Norway; 2 Bergen Addiction Research, Department of Addiction Medicine, Haukeland University Hospital, Bergen, Norway

**Keywords:** Food security, access to healthy foods, general practice, Norway, socioeconomic factors, chronic disease

## Abstract

**Aims:**

We aim to address the knowledge gap surrounding food insecurity in general practice in Norway, focusing on its prevalence among patients, sociodemographic correlates and its relationship with chronic diseases across different age groups.

**Methods:**

This study is cross-sectional, collecting data through 69 general practice clinics in 2022 from patients >18 years old visiting their general practitioner. They answered an anonymous questionnaire with the Cornell–Radimer hunger scale. Questions addressed hunger, concerns about food access, financial difficulties purchasing food, impact on children, patient demographics, children, and use of medications for chronic disease. We present logistic regression models with odds ratios (ORs) with 95% confidence intervals to examine associations between food insecurity and patient characteristics.

**Results:**

Among 2571 invited patients, 81.2% (*n*=2089) participated in the study. Of the participants, 40.1% were considered food insecure. The questions in the Cornell–Radimer hunger scale indicated that most had a mild degree of food insecurity. Food insecurity ranged from 28.9% among those >70 years old to 68.0% among those <30 years old. Food insecurity was associated with age <30 years, being migrant, inversely associated with higher educational levels (OR 0.60, 0.41–0.87) or having own children (OR 0.24, 0.18–0.31). Food insecurity was higher among participants using medications for chronic disease (OR 1.33, 1.05–1.68).

**Conclusions:**

**This study underscores the presence of food insecurity in high-income countries like Norway, particularly among specific groups such as young adults, migrants and patients with chronic diseases. These categories of patient could benefit from screening of food insecurity during medical contact.**

## Key messages

1. Some degree of food insecurity is prevalent among patients in general practice in Western Norway, affecting 40%.
2. Food insecurity is higher among young adults and migrants.
3. Food insecurity is less common among people with higher education.
4. There is an association between food insecurity and chronic diseases.

## Background

Access to adequate food is considered a human right [1]. A key target of the Sustainable Development Goals is to end hunger and ensure access to safe, nutritious and sufficient food by all people by 2030 [2]. Food security is defined as having physical, social and financial access to sufficient, safe and nutritious food to cover dietary needs and food preferences, in order to live an active and healthy life [3]. According to the Food and Agriculture Organization [1], food security is the result of availability of food, access to food, provision stability and, not least, the opportunity to benefit from food involving health, hygiene, clean water, sanitary conditions and knowledge of diet and nutrition [3]. Food insecurity is characterized by limited or uncertain access to nutritionally suitable and safe foods, or the capability to obtain culturally acceptable foods through socially appropriate means [4]. Several studies indicate links between food insecurity and adverse health outcomes such as cardiovascular diseases, hypercholesterolaemia, diabetes, obesity, iron deficiency anaemia, malnutrition, nutritional deficiencies, physical pain, depression and anxiety [5–8]. Food insecure children are more likely to experience poor general health, iron deficiency and poor psychosocial functioning, as well as developmental and behavioural problems in the youngest, and depression and low academic performance in teenagers [7,9,10].

Despite Norway’s having a well-established welfare system, some studies have shown that food insecurity exists both in Norway and in other high-income countries [9]. In 2012, 27–37% of the Nordic population experienced some form of food insecurity. Of these, 1.5–4% experienced a high degree of food insecurity [9]. Typically, these studies focus on populations in vulnerable situations, such as recently arrived refugees or homeless people [8,11]. An assessment of food insecurity in Europe concluded that the lack of knowledge about European food insecurity is alarming and that more empirical research is needed across European countries [12]. Little is known about the level of food insecurity among the general population receiving care and follow-up in primary health care in Norway, or other high-income countries. Research from the US has suggested that food is rarely considered by healthcare personnel, partly due to limited training in management and partly due to lack of time [13]. Thus, it is important to study food insecurity in patients in a general practice setting.

Considering the lack of knowledge about food insecurity in general practice in high income countries such as Norway, the aim of this study was to assess the prevalence of food insecurity in users of primary care service in 2022. Additionally, we investigated associations between food insecurity, sociodemographic factors and chronic diseases.

## Methods

### Study design and study population

The present study is cross-sectional, where data was collected using an anonymous questionnaire. The questionnaires were handed out to patients who were recruited from 69 general practice clinics in February 2022 to October 2022 by 129 medical students in collaboration with clinic staff. The general practice clinics were involved in the training of medical students, and each recruited 20 consecutive and unselected patients over 18 years of age from the waiting room of the general practitioners’ office. The questionnaires were in Norwegian, but English translations were offered for patients who preferred English. The questionnaires were returned in sealed envelopes without identification markers. A total of 2571 patients were invited to the study, out of which 2145 consented to participate and responded to the questionnaire. Questionnaires were returned in a closed envelope and those who did not want to consent could return an empty and unfilled questionnaire in the closed envelope, thus not needing to disclose whether or not they wanted to participate to clinic staff. However, 40 of these respondents were under the age of 18 and were subsequently excluded. Thus, the study included a total of 2105/2571 participants, resulting in a response rate of 83.2%.

### Measures

The questionnaire included the Cornell–Radimer hunger scale. The scale has been evaluated and validated in various populations and settings [14,15]. It presents 10 questions on food insecurity on three levels including the household level, and the level of the person/adult, and their children. The answer alternatives presented in the tool are affirmative (‘often true’ or ‘sometimes true’) or negative (‘never true’). Food insecurity is defined as at least one affirmative answer. The participants also responded to questions about sex, age, children living at home, education, and use of medications (cholesterol-lowering drugs, antihypertensive (blood pressure-lowering drugs), medications for heart disease(s), medication for diabetes/blood sugar levels, obesity, and other medications).

### Statistical analysis

In the analysis, patients who answered affirmatively to any of the questions about medications were considered as having a chronic disease. Further, we use the word immigrant referring to a person born outside of Norway, and grouped countries together into eight groups: Asia, Africa, Latin-America, North America, Oceania, the Nordics, Western Europe, and Eastern Europe. These were further grouped into four categories for the final analysis (Norway; Asia, Africa, Latin-America; Nordics, Western Europe, North America, Oceania; and Eastern Europe).

To avoid including highly correlated variables in regression analyses, we ran a correlation matrix with age, sex, own children, children in the household, country of birth and education, and none of these were correlated above 0.5 (Supplementary material file Table SI online). The strongest correlation was seen between age and having children, or children living at home (0.41; Supplementary Table S1). For selection of variables for regression models, we use the framework by Westreich and Greenland [16]. We used direct acyclic graphs (DAGs) to guide variable selection for each model using DAGitty (https://www.dagitty.net/dags.html) to assist the selection in this study. See further details and direct acyclic graphs’ figures indicating assumed causal inferences for Table II in Supplementary file Figure S1(a) to (f).

To investigate the associations between food insecurity and the patient characteristics, we categorized food insecurity if there was any affirmative answer to any of the 10 questions (see questions 1–10 in the questionnaire, Supplementary file Figure S2(a) to (j )and Figure S3(a) and (b)). To assess associations, we used a series of logistic regression models to assess links between patient characteristics and food insecurity, adjusting for the other included variables (migration and children were adjusted for age; education was adjusted for age, sex, children and migration; and chronic diseases was adjusted for age, migration, sex). We also conducted logistic regression models to assess links between patient characteristics and food insecurity; adjusting for the other included variables and adding an interaction between age and medications for chronic disease. The results are presented as odds ratios (ORs) with 95% confidence intervals. We did not impute or make assumptions to missing values in the regression models, and thus used listwise deletion. For the regression models, between 1933 and 2077 were included in the analyses. All the statistical analyses in this study were performed using Stata SE 18.0 (StataCorp. LLC, College Station, TX, USA).

The study was considered by the Regional Ethical Committee in Western Norway (REK Vest) and was considered ethically acceptable (no. 386617).

## Results

Among the respondents, 1273/2030 (62.7%) were females (Table I), 1523/2024 (75.2%) had children and 1799/2045 (88%) were born in Norway, while 98/2045 (4.8%) were from Asia, Africa, Latin America, 76/2045 (3.7%) from Eastern Europe and 72/2045 (3.5%) were born in other countries. The majority of participants had completed secondary school or university/college. A total of 845/2059 (41%) used medications for chronic diseases. Medications for chronic diseases were used by 364/441 (82.5%) among those above 70 years of age and 17/288 (5.9%) among those 18–30 years of age (Supplementary Table SII).

**Table I.** Descriptive characteristics of the participants (number (*n*) and percentage of valid responses are indicated in parentheses).

	*n* a	%
**Sex (*n*=2030)**		(97.2%)
Female	1273	62.7%
**Age, years (*n*=2077)**		(99.4%)
18–29	294	14.2%
30–39	380	18.3%
40–49	315	15.2%
50–59	309	14.9%
60–69	329	15.8%
70+	450	21.7%
**Having own children (valid *n*=2024, 96.4%)**	1523	75.2%
**Birth country (*n*=2045)**		(97.9%)
Norway	1799	88%
Asia, Africa, Latin America	98	4.8%
Nordics, W Europe, N America, Oceania	72	3.5%
Eastern Europe	76	3.7%
**Education (*n*=2029)**		(97.1%)
Primary and secondary school	180	8.9%
High school or technical school	966	47.6%
University/college	883	43.5%
**Use of medications for chronic disease (*n*=2059)**		(98.6%)
Any medication for chronic disease	845	41%
Statins (reducing cholesterol)	478	23.2%
Antihypertensives	589	28.6%
Other medications for cardiovascular disease	273	13.3%
Medications for diabetes and metabolic disease	221	10.7%
Other medications	1000	48.6%

a Numbers with valid responses vary between variables owing to some not providing valid answers or the responses not being readable.

**Figure 1.**
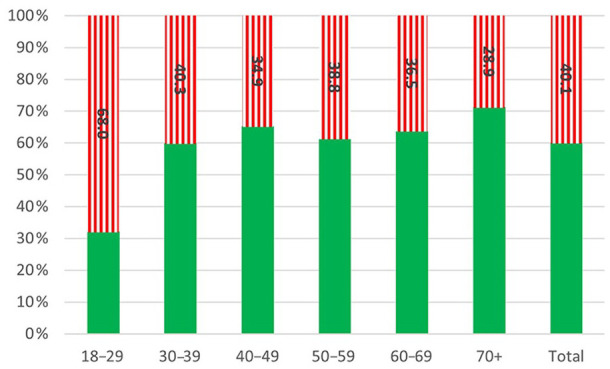
Prevalence of food insecurity in the study population by age group categorized into food secure (green – filled) and food insecure (red – vertical lines).

**Figure 2.**
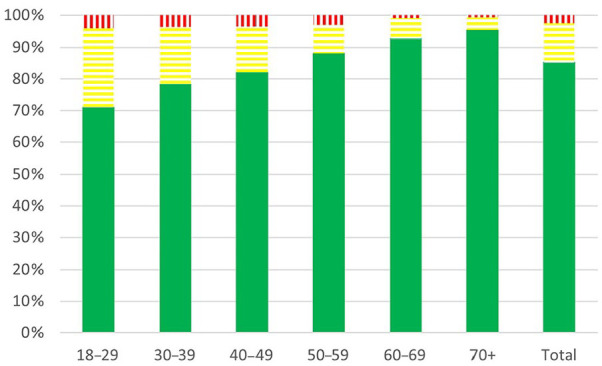
Worries of not having enough food in each age group categorized into never (green – filled), sometimes (yellow – horizontal lines) and often (red – vertical lines).

Of the respondents, 833/2077 (40.1%) were found to be food insecure (Figure 1). The most frequently reported dimension of food insecurity was related to food availability (Supplementary file Figure S2(a) to (j)). Of the 833 found to be food insecure, only 25 (3.0%) answered that they were often hungry, while 256 (30.7%) reported not affording to eat well. Out of 1526 respondents with children, 15 (0.9%) indicated that they were unable to procure food for their children. In the youngest age group (18–29 years), 85/293 (29.0%) respondents reported worries about not having enough food, while the age group over 70 years old reported this rarely (21/446, 4.7%) (Figure 2). In the adults under 30 years old not receiving medications for cardiovascular or metabolic diseases, 68.0% reported food insecurity, compared with 28.9% in those aged 70 years or more.

Being older than 30 years was negatively associated with being food insecure (for >70 years compared with < 30: OR 0.19, 0.14–0.26) (Table II). No significant difference in food insecurity was present between sexes. Furthermore, food insecurity was associated with being migrant (for Asia, Africa, Latin America: OR 2.06, 1.35–3.16). Food insecurity is also inversely associated with having children (OR 0.24, 0.18–0.31). Food insecurity was less common among people with higher education (university/college) levels (OR 0.60, 0.41–0.87) when compared with people with lower level of education, elementary and secondary school. An association was seen between food insecurity and chronic disease in the age groups under 50 years (only significant for the age range 30–39 years of age), but not among the older adults (Supplementary Table SIII). The adjusted OR for food insecurity associated with use of medications for chronic diseases was higher when adjusting for variables such as age, because chronic diseases are strongly associated with increasing age, which is also strongly inversely associated with food insecurity.

**Table II.** Prevalence of food insecurity in various groups and association of food insecurity with socio-demographic characteristics and medications for chronic disease presented with odds ratios and 95% confidence intervals as well as average marginal effects (using logistic regression models).

Factor associated with food insecurity	Prevalence	Adjusted model	Marginal effects
		OR (95% CI)	dy/dx (95% CI)
**Age, years**			
18–29	200 (68%)	1	1
30–39	153 (40%)	**0.32 (0.23–0.44)**	−0.28 (−0.35; −0.21)
40–49	110 (35%)	**0.25 (0.18–0.35)**	−0.33 (−0.41; −0.26)
50–59	120 (39%)	**0.30 (0.21–0.42)**	−0.29 (−0.37; −0.22)
60–69	120 (36%)	**0.27 (0.19–0.38)**	−0.32 (−0.39; −0.24)
70+	130 (29%)	**0.19 (0.14–0.26)**	−0.39 (−0.46; −0.32)
**Sex**			
Female	515 (40%)	1	1
Male	302 (40%)	0.98 (0.81–1.17)	−0.01 (−0.05; 0.04)
**Do you have own children?**			
No	341 (69%)	1	1
Yes	465 (31%)	**0.24 (0.18–0.31)**	−0.34 (−0.40; −0.28)
**Birth country**			
Norway	683 (38%)	1	1
Asia, Africa, Latin America	55 (56%)	**2.06 (1.35–3.16)**	0.17 (0.07; 0.27)
Nordics, W Europe, N America, Oceania	40 (56%)	**2.02 (1.24–3.29)**	0.16 (0.05; 0.28)
Eastern Europe	38 (50%)	**1.69 (1.05–2.72)**	0.12 (0.01; 0.23)
**Highest completed education**			
Primary and secondary school	75 (42%)	1	1
High school or technical school	413 (43%)	0.90 (0.63–1.29)	−0.02 (−0.10; 0.06)
University/college	313 (35%)	**0.60 (0.41–0.87)**	−0.11 (−0.18; −0.03)
**Medications for chronic diseases**			
No	517 (43%)	1	1
Yes	311 (37%)	**1.33 (1.05–1.68)**	0.06 (0.01; 0.11)

Models are structured based on variable selection using the framework of Westreich and Greenland with adjustments according to assumed causal models (see Supplementary Figure S1(a) to (f) for details). Age and sex are not adjusted. Having children and migration are both adjusted for age. Education is adjusted for age, sex, children, migration. Medications for chronic disease is adjusted for age, sex and migration. Average marginal effects for factor levels presented as dy/dx (the discrete change from the base level).

OR: odds ratio; CI: confidence interval

## Discussion

Some degree of food insecurity was common among patients visiting their general practitioner, being reported among 40.1% of the participants. Young age, being immigrant, and having chronic diseases were the factors most strongly associated with food insecurity.

Another consumer oriented study from Norway using another tool to measure food insecurity estimated the prevalence of food insecurity in Norway to be 3% [17]. The discrepancy with the present study might be due to the different tool and sample of population, and most of their findings are approximately 10 years older than the data from this study. However, the identified risk categories are similar to the ones found in the present study. The need to buy cheap food options to afford other necessities was reported by approximately 40% in the consumer study. Based on reporting from each of the items in the Radimer–Cornell questionnaire in this study, food insecurity identified with the tool we used includes a range in degrees of food insecurity, with most of the identified food insecurity being in the milder part of the spectrum.

Individuals with higher educational levels had slightly lower prevalence of food insecurity compared with those in the lower education categories such as primary and secondary school and high school/technical school. Despite that one should be careful of comparing ORs between different studies [18], it is worth noting that previous research also points towards a strong association between low educational level and food insecurity [4,19]. It is important to acknowledge that variations in the sample characteristics, study design and contextual factors may contribute to these divergent results.

Interestingly, having children was the variable that was strongest inversely associated with food insecurity. Patients with children worried about food much less frequently compared with those without children. Another study analysing food insecurities in the Nordic countries found a higher prevalence of food insecurity in single parents [4]. In a study from Central and South America, families with children reported greater concerns about food compared with those without children [20]. The results from this study with a low prevalence of food insecurity among families with children might be explained by Norway’s comprehensive social assistance and support system, which prioritizes the well-being of children.

People born in Norway had almost half the OR of being food insecure compared with migrants. Overall, the literature suggests high rates of food insecurity among immigrant populations [11]. In this study, it is unfortunately not possible to distinguish between recent and long-term migrants or reason for migration. Important barriers to food security in recent migrants include language difficulties and limited knowledge of available food resources, in addition to economic reasons, as well as difficulty accessing culturally appropriate foods from the migrants’ perspective [21]. Some scoping reviews among immigrants suggest that food insecurity is linked to poor nutrition [11,22].

Among different age groups, there was a notable difference in the prevalence of food insecurity, with younger individuals less than 30 years of age being much more susceptible to experiencing food insecurity compared with adults above 30 years of age. In the adults under 30 years not receiving medications for cardiovascular or metabolic diseases, more than two-thirds reported food insecurity, compared with less than one-third of those aged 70 years or more. The findings from this study are consistent with a study which observed a higher prevalence of food insecurity among young people in Nordic countries [9]. Risk factors that may be more common in young adults, such as unstable housing situations, living alone and low income, were identified as risk factors in several studies [23,24]. Addressing food insecurity in young adults is of paramount importance, as it has been linked to mental health problems, higher risk of suicide, and substance use [5]. On the other hand, it is important to note that some studies indicate that restricting food consumption could be a maladaptive coping strategy of mental health difficulties rather than a symptom of food insecurity in some individuals [25].

The present study revealed a significant association between food insecurity and chronic disease, particularly in younger adults. Worthy of concern, food insecurity could exacerbate chronic diseases. Having more information about the relationship between chronic diseases and food insecurity may lead health providers to involve social service professionals as well. One French study offers resources for healthcare professionals to engage in this topic in a secure and healthy way. By educating themselves on the impact of food insecurity on chronic diseases, healthcare providers can better understand the needs of their patients and address the underlying causes of health disparities [14]. Additionally, research on food security and obesity in the United States found that regardless of ethnicity, those experiencing food insecurity without hunger had a higher prevalence of obesity compared with their food secure counterparts [26]. In addition to consuming low-cost, energy-dense foods, individuals facing substantial food insecurity may exhibit a cyclic pattern of ‘feasting and famine’ due to the timing of their monthly lump sums of public assistance. The pattern involves excessive food intake when resources are available, followed by periods of fasting when resources are depleted. Such a feast–famine cycle has been linked to a biological predisposition to obesity and diabetes in observational studies and animal models [27]. The relationship between food insecurity, dietary patterns and chronic diseases such as obesity, diabetes and cardiovascular disease shows a complex interplay [6].

It is important to note that the association between food insecurity and chronic disease was observed only among younger individuals, suggesting that other factors may play a role in the association between chronic disease and food insecurity. Further research is needed to explore these factors and gain a better understanding of the intricate interplay between food insecurity, chronic disease and age across diverse populations. Further, the findings from this study have important implications for public health interventions and targeted support. The findings of this study suggest a need to tailor interventions addressing food insecurity to meet the needs of young adults and individuals in their 20s, and migrants. Such interventions may involve ensuring access to nutritious food and providing holistic management of chronic diseases. Stronger involvement of dietitians and nutritionists in primary health care might also be a strategy to increase focus on food and food insecurity.

This study has several strengths and limitations. The Cornell–Radimer hunger scale offers a comprehensive assessment by considering various dimensions of food insecurity, encompassing aspects such as the frequency of food shortages as well as concerns about food and disrupted eating patterns [28]. However, as a self-reported tool, responses are subject to individual perceptions, potentially introducing biases. For most of the analyses of this study, we have dichotomized the multidimensional food insecurity instrument to operationalize food insecurity, which limits nuances such as different degrees of food insecurity in most analyses. There is also a range of alternatives to the Cornell–Radimer hunger scale, including the Core Food Security Measurement/Household Food Security Survey Module and the Food Insecurity Experience Scale (https://www.fao.org/in-action/voices-of-the-hungry/fies/en/) [29], both with many similarities to the tool we used and also validated in a range of contexts. Furthermore, the present study is cross-sectional, which limits its ability to capture changes over time and understand the long-term effects of food insecurity, or to causally interpret the observed associations. Nevertheless, in identifying groups at risk, the study is still important. It is possible that people may tend to report food security in a way that is socially acceptable [30]. Anonymous reporting probably limited this tendency. The sampling is from a clinical context with consecutive sampling, but is not randomly assigned, which might contribute to overestimation of the prevalence of food insecurity. There could be some degree of clustering between participants as they were recruited from 69 clusters. As we do not have clinic linkages to each of the questionnaires, accounting for clustering design effects is not possible. The estimates of this study might thus appear minimally more precise than if the clustering had been taken into consideration. Further, we do not have information on the number of patients linked to each of the clinics, and the number of patients recruited from each of the clinics could have been made proportional to clinic size instead of fixed to 20 patients. However, this would have provided logistical difficulties with uncertainty in degree of gain in representativeness. The category of chronic diseases was operationalized through use of medications for cardiovascular and metabolic diseases. There are several chronic diseases not covered within this definition such as chronic obstructive pulmonary diseases.

The analysis of this study highlights the necessity for further information to gain a deeper understanding of the underlying factors driving food insecurity. To address this, we propose that future studies collect additional data regarding the length of stay in Norway, reason for migration, income levels and household status. The sample is restricted to the people visiting their general practitioner, excluding individuals who experience language or cultural barriers or communication difficulties; on the other side, the sample represents several ethnicities, a range of ages, and educational background. As some respondents had missing information on some key variables and we did not impute or assume numbers when missing, up to 7% of the sample were not included in some of the regression models. We provided questionnaires in both Norwegian and English, with 5% of the questionnaires being in English. For immigrants who did not communicate in Norwegian or English it was likely to be more difficult to respond, and based on the findings from this study we could expect the proportion of food insecurity among these to be higher.

## Conclusion

This study highlights the presence of food insecurity in patients in general practice in Norway, particularly among specific groups such as young adults, migrants and patients with chronic diseases. Despite Norway’s being a welfare state, groups such as young adults and immigrants born outside of Norway were associated with food insecurity. Furthermore, we found an association between chronic disease and food insecurity in the younger age groups. These categories of patient could benefit from screening of food insecurity during healthcare contacts as part of a holistic care approach, considering the importance of food security for health and well-being. By understanding the magnitude of food insecurity in high-income countries and implementing appropriate food policies, it might be possible to limit food insecurity and mitigate the adverse effects of food insecurity.

## Supplemental Material

sj-docx-1-sjp-10.1177_14034948241278781 – Supplemental material for Food insecurity in Norway: A cross-sectional study among patients visiting their general practitioner Supplemental material, sj-docx-1-sjp-10.1177_14034948241278781 for Food insecurity in Norway: A cross-sectional study among patients visiting their general practitioner by Noemi Cioffi, Esperanza Diaz, Elisabeth M. Strømme, Bjørn Bjorvatn, Thomas Mildestvedt and Lars T. Fadnes in Scandinavian Journal of Public Health
