# Supplementary material for: Food insecurity in Norway: A cross-sectional study among patients visiting their general practitioner
Source: Scand J Public Health. 2024 Sep 26;54(1):8–15. doi: 10.1177/14034948241278781 (PMC12858646; doi:10.1177/14034948241278781)
Supplement: sj-docx-1-sjp-10.1177_14034948241278781 – Supplemental material for Food insecurity in Norway: A cross-sectional study among patients visiting their general practitioner [file sj-docx-1-sjp-10.1177_14034948241278781.docx]

Supplementary for food insecurity in Norway: a cross-sectional study among patients visiting their general practitioner.

**Content:**

**Figure S1 (a-f):** Direct acyclic graphs indicating assumed causal inferences for table 2 (page 2-7)

**Figure S2 (a-h):** Response to each of ten questions in the food insecurity questionnaire (page 8-17)

**Figure S3 (a-b):** Answers to question 1 to 10 presented by sex. (page 18-19)

**Table S1:** Correlation matrix (page 20)

**Table S2:** Descriptive characteristics of the participants and proportion within each strata using medications for chronic diseases (number [n] and percentage of valid responses are indicated in parentheses).

**Table S3:** Food insecurity in various groups as well as interaction between age and medication for chronic diseases and correlates of food insecurity presented with odds ratios (OR) and 95% confidence intervals (CI) (page 21).

**Figure S1a-f: Direct acyclic graphs indicating assumed causal inferences for table 2**

A: Outcome food insecurity. Exposure: Age. No adjustments.


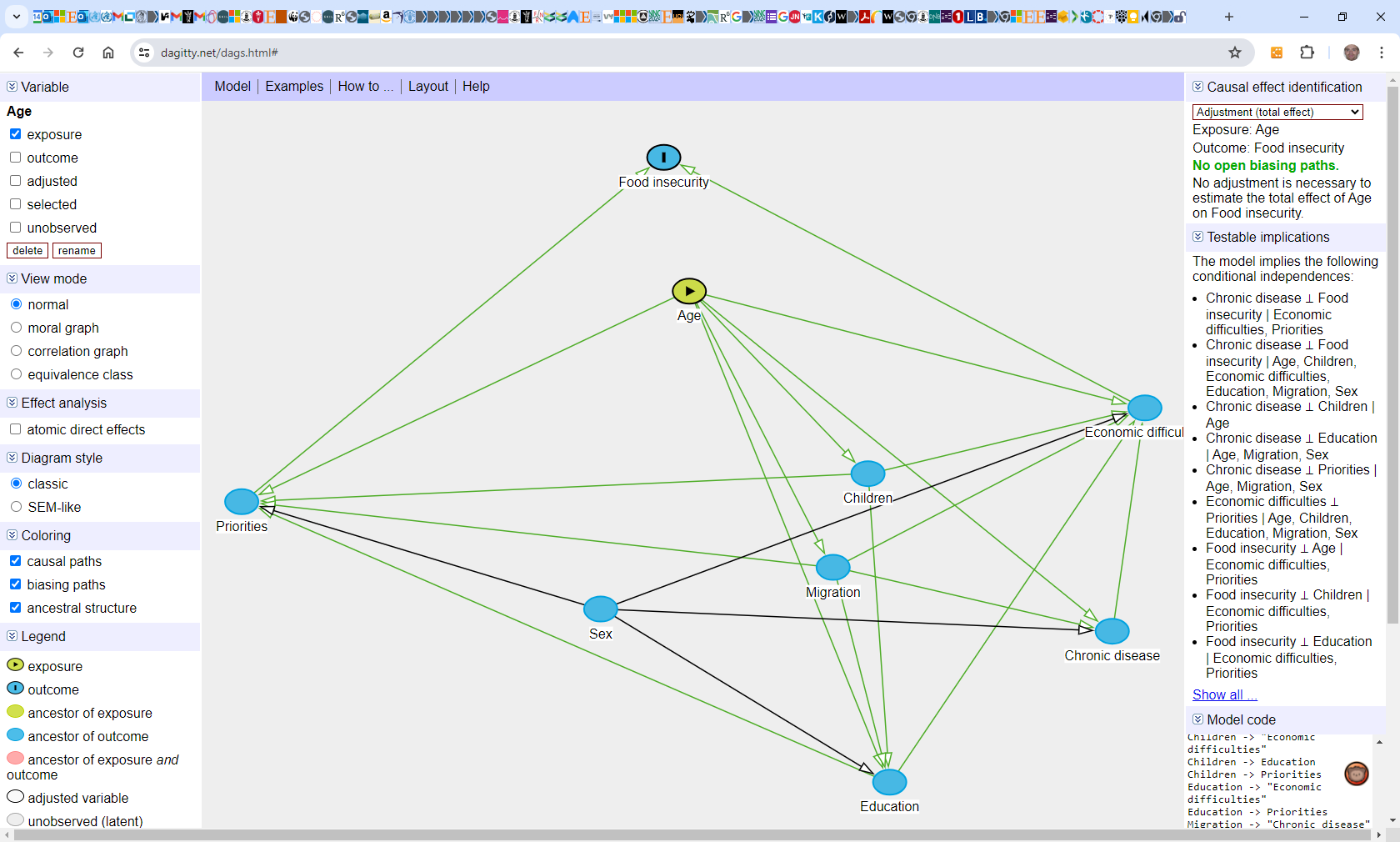


B: Outcome food insecurity. Exposure: Sex. No adjustments.


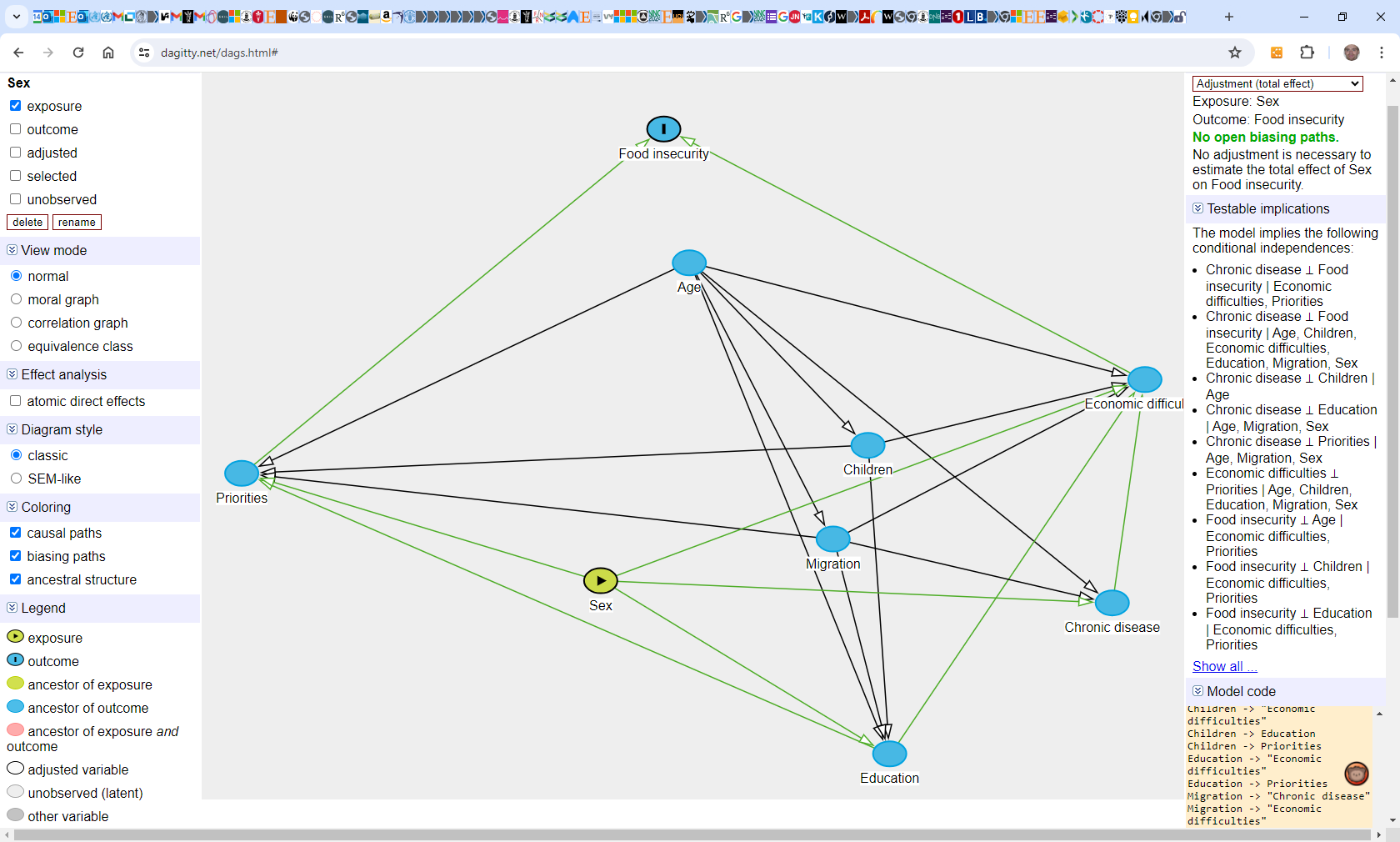


C : Outcome food insecurity. Exposure: Children. Adjustment for age.


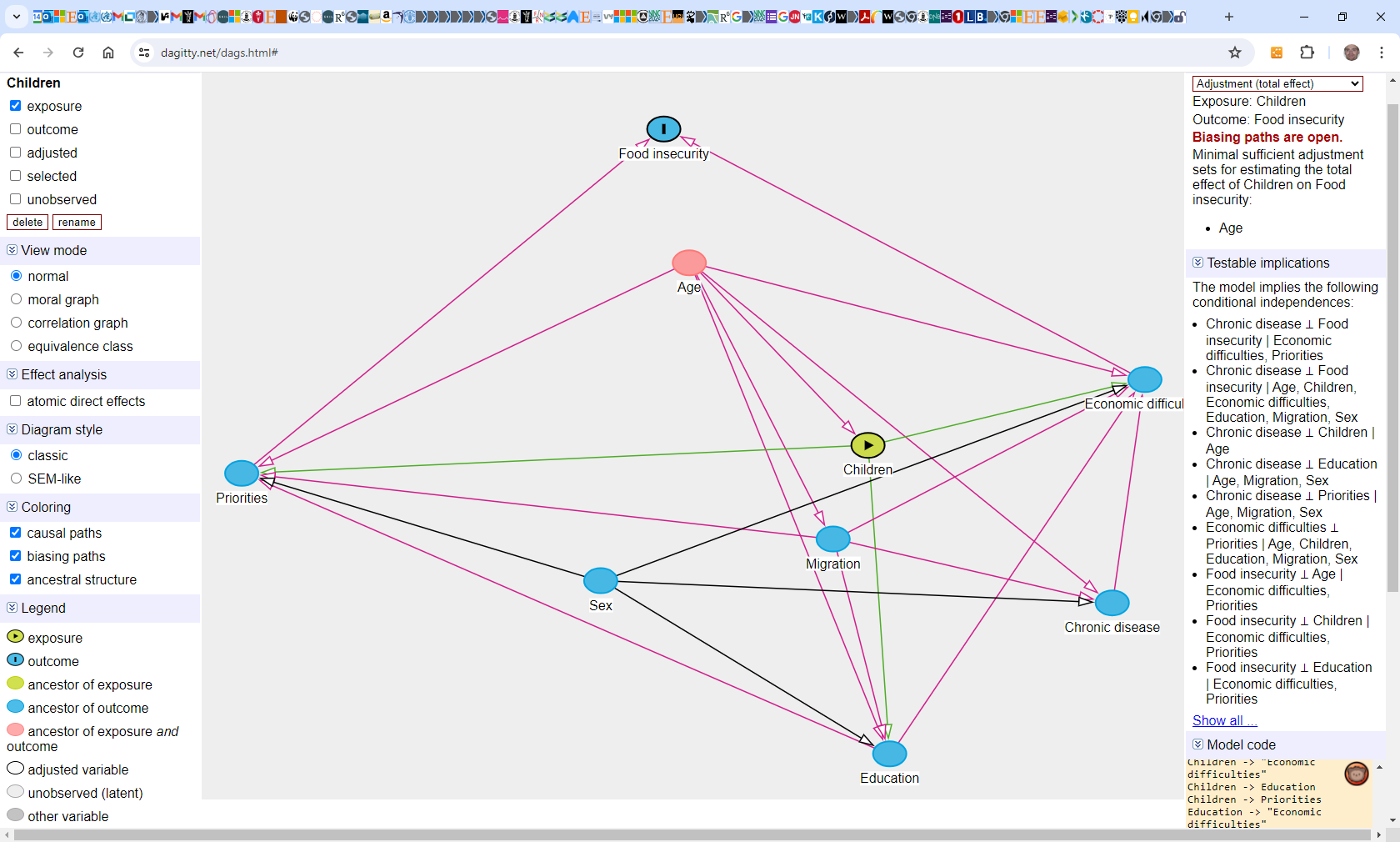


D: Outcome food insecurity. Exposure: Migration. Adjustment for age.


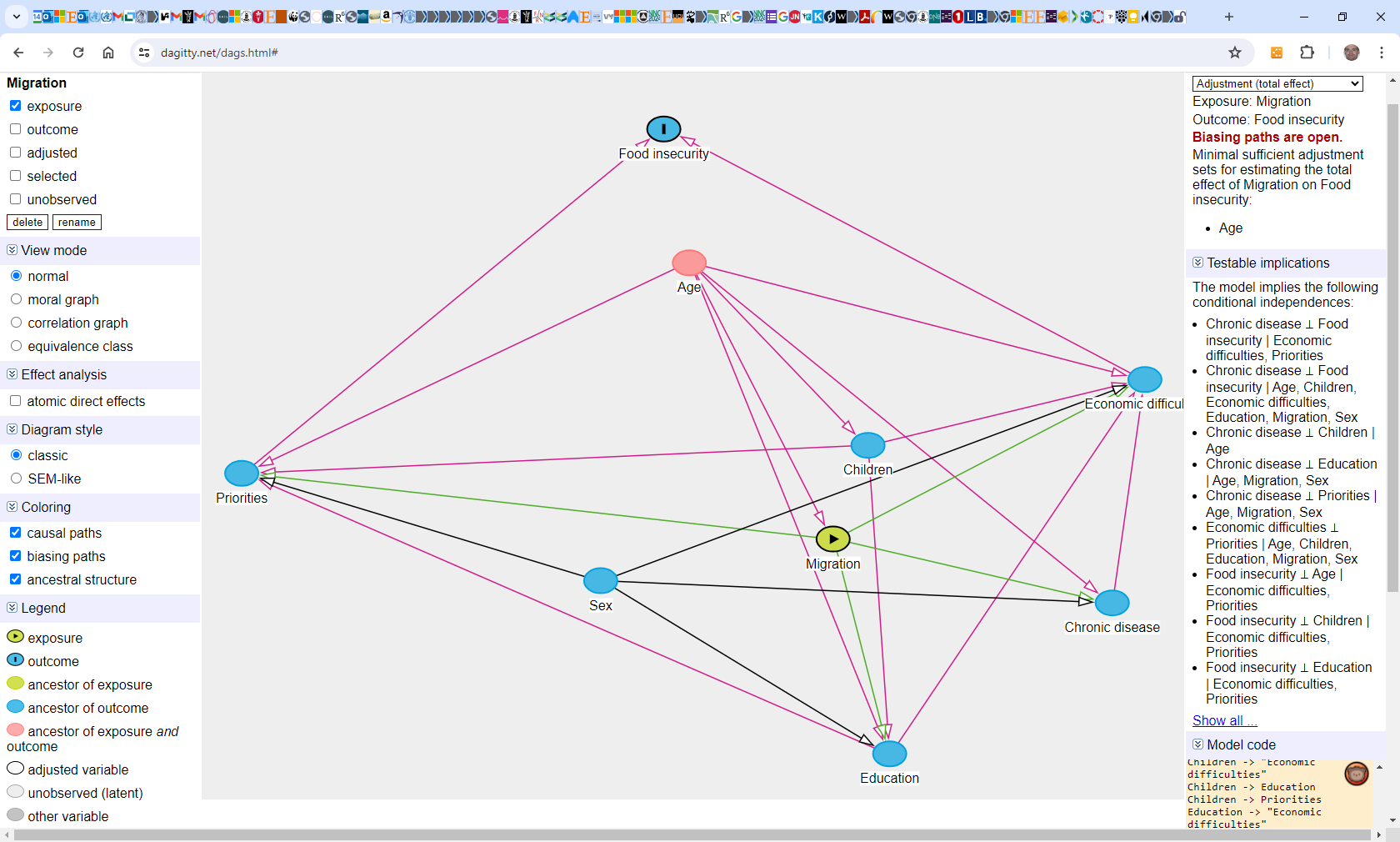


E: Outcome food insecurity. Exposure: Education. Adjustment for age, sex, children, migration.


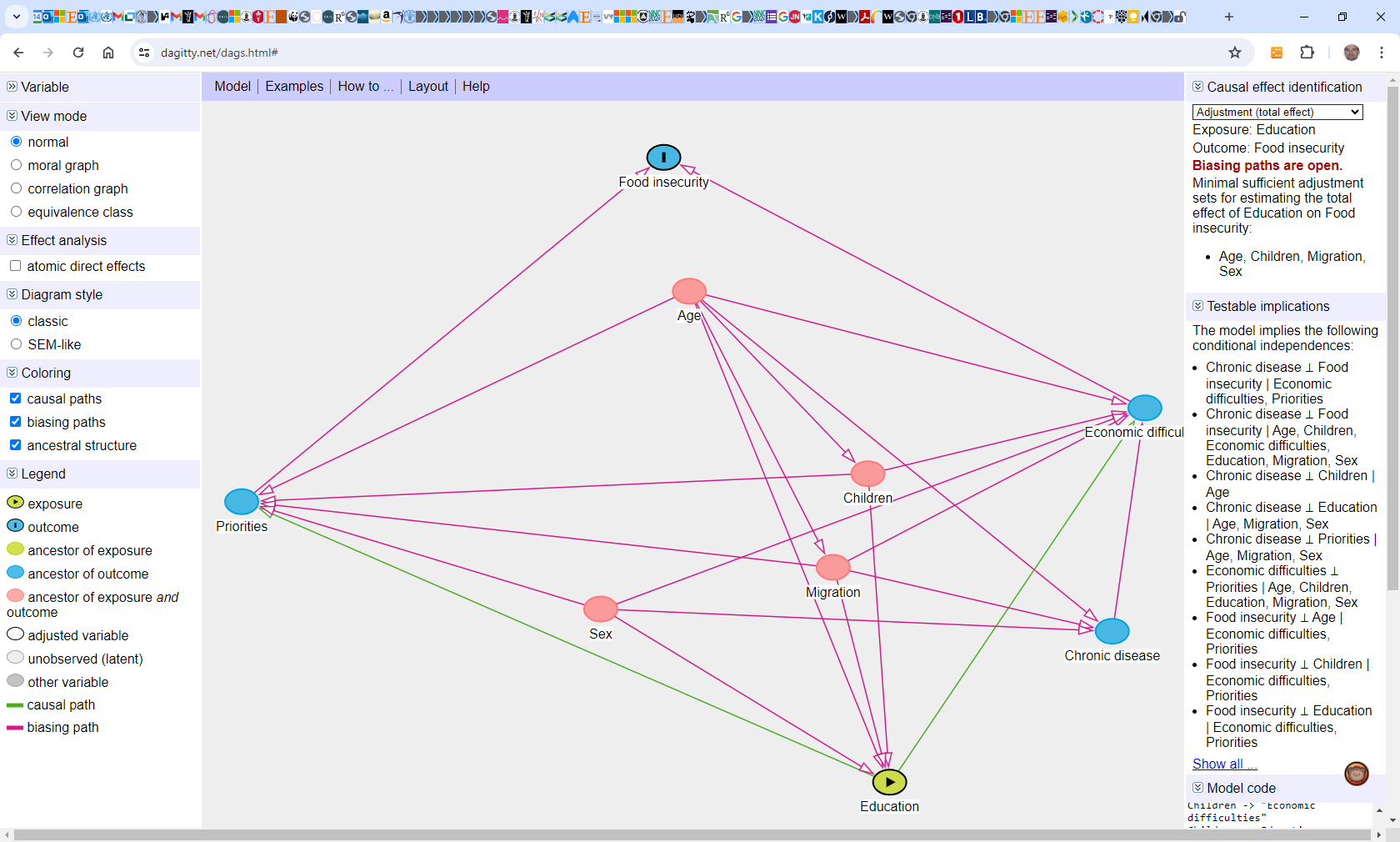


F: Outcome food insecurity. Exposure: Chronic disease. Adjustment for age, migration, sex.


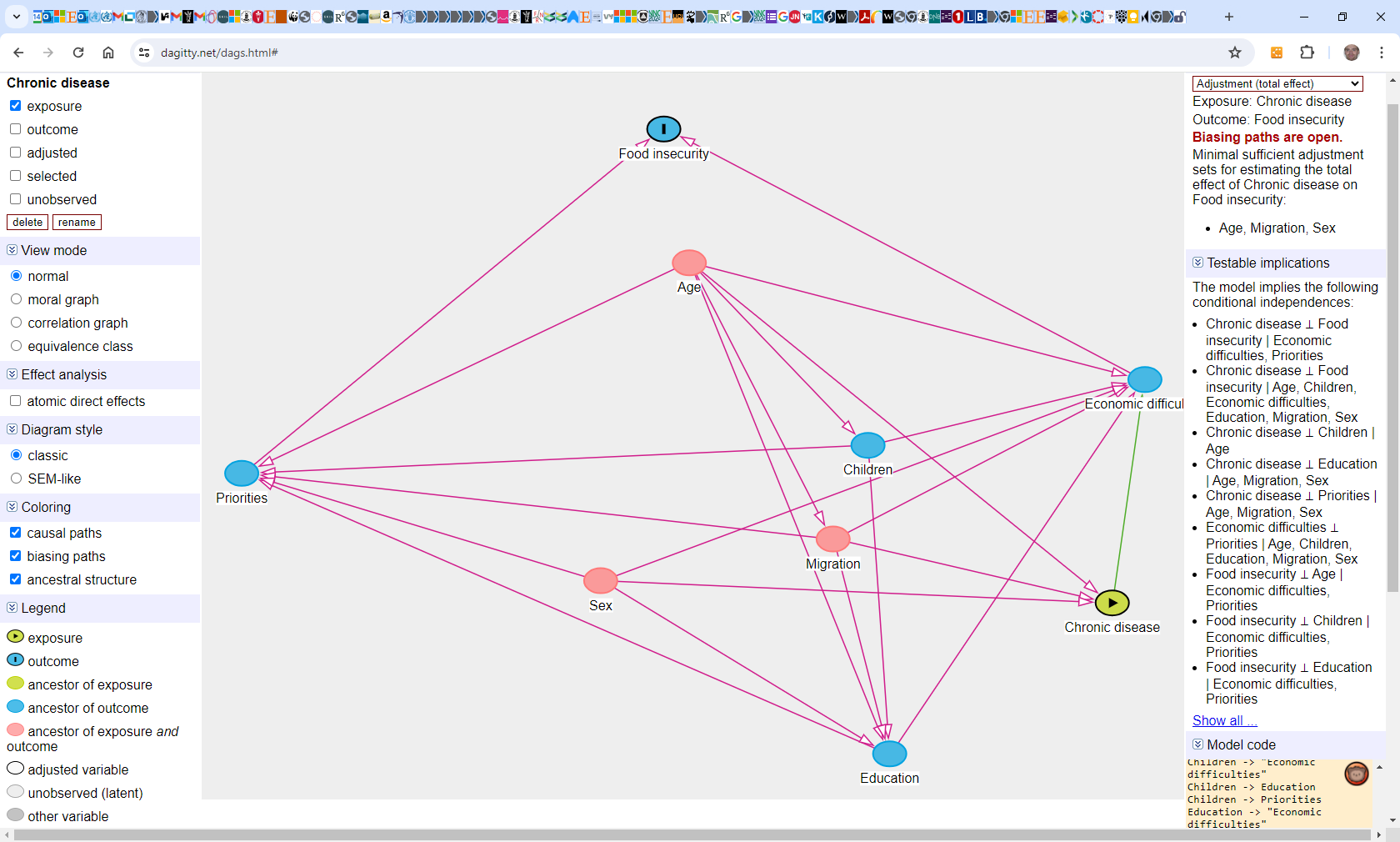


**Figure S2a-j:** Response to each of the ten questions in the food insecurity questionnaire

1. answers to question 1, by age: "I worry that I will run out of food before I do get money to buy more"

The six age groups are presented here. Green on the bottom is for none/never, yellow in the middle is for some/sometimes and red on top is for substantial/often.

**Figure S2b**

1. answers to question 2, by age: "We eat the same thing several days in a row because we have few food available and do not have money to buy more"

The six age groups are presented here. Green on the bottom is for none/never, yellow in the middle is for some/sometimes and red on top is for substantial/often.

**Figure S2c**

1. answers to question 3, by age: "The food I bought was not sufficient and I did not have money to buy more"

The six age groups are presented here. Green on the bottom is for none/never, yellow in the middle is for some/sometimes and red on top is for substantial/often.

**Figure S2d**

1. answers to question 4, by age: "I ran out of the food I needed to prepare one meal and had no money to buy more"

The six age groups are presented here. Green on the bottom is for none/never, yellow in the middle is for some/sometimes and red on top is for substantial/often.

**Figure S2e**

1. answers to question 5, by age: "I'm often hungry, but I don't eat because I can’t afford buying enough food"

The six age groups are presented here. Green on the bottom is for none/never, yellow in the middle is for some/sometimes and red on top is for substantial/often.

**Figure S2f**

1. answers to question 6, by age: "I eat less than I think I should because I don't have enough money for food"

The six age groups are presented here. Green on the bottom is for none/never, yellow in the middle is for some/sometimes and red on top is for substantial/often.

**Figure S2g**

1. answers to question 7, by age: "I can't afford to eat well"

The six age groups are presented here. Green on the bottom is for none/never, yellow in the middle is for some/sometimes and red on top is for substantial/often.

**Figure S2h**

1. answers to question 8, by age: "I cannot give my child/children the food that I think they should get, because I can't afford it"

The six age groups are presented here. Green on the bottom is for none/never, yellow in the middle is for some/sometimes and red on top is for substantial/often.

**Figure S2i**

1. answers to question 9, by age: "My child/children des/do not eat enough because I cannot afford enough food at all"

The six age groups are presented here. Green on the bottom is for none/never, yellow in the middle is for some/sometimes and red on top is for substantial/often.

**Figure 2j**

1. answers to question 10, by age: "I know that my child/children are sometimes hungry, but I simply cannot afford more food"

The six age groups are presented here. Green on the bottom is for none/never, yellow in the middle is for some/sometimes and red on top is for substantial/often.

**Figure S3a**: Answers to question 1 to 10 presented by sex. This is for females.

Green on the bottom is for none/never, yellow in the middle is for some/sometimes and red on top is for substantial/often.

**Figure S3b:** Answers to question 1 to 10 presented by sex. This is for males.

Green on the bottom is for none/never, yellow in the middle is for some/sometimes and red on top is for substantial/often.

**Table S1:** Correlation matrix

|  | Age | Sex | Having children | Children at home | Country of birth | Education |
| --- | --- | --- | --- | --- | --- | --- |
| Age | — |  |  |  |  |  |
| Sex | 0.1 | — |  |  |  |  |
| Having children | 0.5 | -0.1 | — |  |  |  |
| Children at home | -0.3 | -0.1 | 0.4 | — |  |  |
| Country of birth | -0.1 | 0 | 0 | 0.1 | — |  |
| Education | -0.1 | 0 | 0 | 0.2 | 0 | — |

**Table S2:** Descriptive characteristics of the participants and proportion within each strata using medications for chronic diseases (number [n] and percentage of valid responses are indicated in parentheses).

|  | **n** | **%** | **Chronic disease** |
| --- | --- | --- | --- |
| **Sex (n=2030)** |  | (97.2%) |  |
| Female | 1273 | 62.7% | 449/1255 (35.8%) |
|  |  |  |  |
| **Age (n=2077)** |  | (99.4%) |  |
| 18-29 | 294 | 14.2% | 17/288 (5.9%) |
| 30-39 | 380 | 18.3% | 47/376 (12.5%) |
| 40-49 | 315 | 15.2% | 89/312 (28.5%) |
| 50-59 | 309 | 14.9% | 128/306 (41.8%) |
| 60-69 | 329 | 15.8% | 193/324 (59.6%) |
| 70+ | 450 | 21.7% | 364/441 (82.5%) |
|  |  |  |  |
| **Having own children (valid n=2024, 96.4%)** | 1523 | 75.2% | 700/1500 (46.7%) |
|  |  |  |  |
| **Birth country (n=2045)** |  | (97.9%) |  |
| Norway | 1799 | 88% | 757/1778 (42.6%) |
| Asia, Africa, Latin-America | 98 | 4.8% | 26/94 (27.7%) |
| Nordics, Western Europe, North America, Oceania | 72 | 3.5% | 25/71 (35.2%) |
| Eastern Europe | 76 | 3.7% | 18/73 (24.7%) |
| **Education (n=2029)** |  | (97.1%) |  |
| Primary & secondary school | 180 | 8.9% | 113/177 (63.8%) |
| High school or technical school | 966 | 47.6% | 431/952 (45.3%) |
| University/college | 883 | 43.5% | 277/874 (31.7%) |

**Table S3:** Food insecurity in various groups as well as interaction between age and medication for chronic diseases and correlates of food insecurity presented with odds ratios (OR) and 95% confidence intervals (CI).

| **Factor correlated with food insecurity** | | **Adjusted model*** |
| --- | --- | --- |
|  |  | OR 95% CI |
| **Age** | |  |
|  | 18-29 | 1 |
|  | 30-39 | 0.32 (0.23-0.44) |
|  | 40-49 | 0.25 (0.18-0.35) |
|  | 50-59 | 0.3 (0.21-0.42) |
|  | 60-69 | 0.27 (0.19-0.38) |
|  | 70+ | 0.19 (0.14-0.26) |
| **Sex** | |  |
|  | Female | 1 |
|  | Male | 0.98 (0.81-1.17) |
| **Do you have own children?** | |  |
|  | No | 1 |
|  | Yes | 0.24 (0.18-0.31) |
| **Birth country** | |  |
|  | Norway | 1 |
|  | Asia, Africa, Latin-America | 2.06 (1.35-3.16) |
|  | Nordics, Western Europe, North America, Oceania | 2.02 (1.24-3.29) |
|  | Eastern Europe | 1.69 (1.05-2.72) |
| **Highest completed education** | |  |
|  | Primary & secondary school | 1 |
|  | Highschool or technical school | 0.9 (0.63-1.29) |
|  | University/college | 0.6 (0.41-0.87) |
| **Medications for chronic diseases** | |  |
|  | No | 1 |
|  | Yes | 1.17 (0.67-2.06) |
| **Interaction age*chronic diseases medications** | |  |
|  | 18-29 with medication | 1.92 (0.48-7.75) |
|  | 30-39 with medication | 2.73 (1.13-6.59) |
|  | 40-49 with medication | 1.85 (0.86-3.99) |
|  | 50-59 with medication | 0.59 (0.28-1.23) |
|  | 60-69 with medication | 0.91 (0.44-1.9) |
|  | 70+ with medication | 1 |

* Models are structured based on variable selection using the framework of Westreich and Greenland with adjustments according to assumed causal models (see Supplementary Figure S1a-f for details). Age and sex are not adjusted. Having children and migration are both adjusted for age. Education is adjusted for age, sex, children, migration. Medications for chronic disease and its interaction with age are adjusted for age, sex, and migration and the interaction between medication and age.
